# Phytochemical and Safety Evaluation of Hydroethanolic Leaf Extract of* Tecoma stans* (L.) Juss. ex Kunth

**DOI:** 10.1155/2019/7417624

**Published:** 2019-04-08

**Authors:** Christopher Larbie, Christabel Owusu Nyarkoh, Clement Owusu Adjei

**Affiliations:** Department of Biochemistry and Biotechnology, Kwame Nkrumah University of Science and Technology, Kumasi, Ghana

## Abstract

*Tecoma stans* (yellow bell) is a popular ornamental plant mostly found in the tropical regions. It is reported to have significant pharmacological activity and has gained attention by natives of various cultures. This study focused on the phytoconstituents screening, antioxidant activity, and heavy metal analysis as well as the acute and subchronic toxicity of the hydroethanolic leaf extract (TSE) using standard methods. The presence of flavonoids, alkaloids, cardiac glycosides, saponins, coumarins, and terpenoids in the raw leaf of the plant was observed while the hydroethanolic extract contained coumarins, saponins, cardiac glycosides, and flavonoids. The DPPH percentage scavenging activity of the crude extract was 64.32% while the fractions, ethyl acetate (55.26%), methanol (60.72%), and hydro (36.97%.), gave varying activities. The UV-Vis and FT-IR indicated the presence of alcohols, phenols, alkanes, alkenes, carbonyls (general), aliphatic amines, aromatics, ketones, ethers, esters, carboxylic acids, alkyl halides, saturated aliphatic acids, 1° and 2° amines, amides, and *α*,*β*-unsaturated aldehydes. The heavy metal analysis showed a high level of iron (Fe) and zinc (Zn) in the raw leaf. The median acute toxicity (LD_50_) of the extract was determined to be <5000mg/kg body weight in mice. Subchronic use for 28 days resulted in significant weight gain, reduction in platelet levels, decrease in WBCs, and increase in blood glucose compared to the normal. TSE caused no adverse effects on vital organs. No mortality was recorded. The hydroethanolic extract of* T. stans* could therefore be considered safe in moderate doses.

## 1. Introduction

Plants have been used for the treatment of several diseases for so many years now before the introduction of orthodox drugs which have their bioactive constituents synthesized from plants with modifications. Antibacterial, antifungal, antimicrobial, and anticancer properties are all found as a result of the secondary metabolites of plants [1–3]. In recent years, there has been an increasing utilisation of medicinal plant because of the conviction that these plants are characteristically innocuous. Be that as it may, numerous adverse responses to medicinal plants have been reported, and with the worldwide ascent in their use, the safety and efficacy of medicinal plants have become a general concern. As indicated by WHO, out of the numerous plants utilised for their medicinal purposes all over the world, just a few have been examined scientifically [4]. This calls for thorough assessment of medicinal plants to guarantee their quality, efficacy, and safety preceding their acceptance and use [5].

*Tecoma stans *(L.) Juss. ex Kunth, commonly known as yellow bell, belongs to the family Bignoniaceae and is distributed worldwide, mostly growing in the tropical and subtropical countries. Phytochemical studies on the plant have shown the presence of alkaloids, tecomanine, iridoid glycosides, lapachol, and other primary and secondary plant metabolites such as sugars, triterpenoids, sterols, and phenolics. All these compounds have been identified in the whole plant at different concentrations [6, 7]. Almost each part of the plant is of therapeutic value. Its leaves show anthelmintic activity, antispasmodic effect, antibacterial activity, anticancer activity, and wound healing property [8, 9]. Flowers showed antidiabetic and anticancer activity while roots showed antibacterial activity. Aerial parts showed antioxidant while the bark possesses wound healing activity [10].

Nonetheless, the toxicity and efficacy of the leaf extract have not been scientifically evaluated. Primary studies in this manner are required to help in clinical work as a gauge for future research. The current study focused on determining the phytochemical constituents, antioxidant activity, heavy metal concentration, and UV-Vis and FT-IR spectra of the hydroethanolic leaf extract of* T. stans* cultivated in Ghana as well as its acute and subchronic toxicity in animals.

## 2. Materials and Methods

### 2.1. Plant Extraction and Fractionation

The leaves of* T. stans* were collected from the lawns behind the Chemistry Building, KNUST Kumasi Campus, before 9:00 am on the 1st and 8th of September, 2017. The leaves were washed and shade-dried for 3 weeks and milled coarsely. A 500 g amount of plant material was extracted twice by percolation using 5 L of 50% of hydroethanolic solution (50:50 v/v ethanol: water). The resulting extracts were pulled together and concentrated using Rotary evaporator at 60°C done under pressure. The extract was freeze-dried to obtain the powdered form of the* T. stans *extracts (TSE).

### 2.2. Sequential Fraction of the Extract

The crude extract was subjected to sequential fractionation with organic solvents of increasing polarity: petroleum ether, ethyl acetate, and methanol. These solvents were used in the fractionation of TSE using a separating funnel. About thirty (30) grams of TSE was weighed into the separating funnel and 300 mL of the solvent was added and shaken vigorously and extracted for 48 hours. The residue was taken out, dried to evaporate the solvent, and then extracted with subsequent solvent. The residue left was labelled as hydro fraction. The four fractions obtained were concentrated and air-dried. They were designated as petroleum ether, ethyl acetate, and methanolic and hydroethanolic fractions.

### 2.3. Phytochemical Screening

The presence of the secondary metabolites of the* T. stans* extract which includes glycosides, tannins, coumarins, saponins, flavonoids, sterols, and terpenoids was tested for using standard methods described previously [11–13].

### 2.4. Determination of DPPH Scavenging Activity

The antioxidant activity of the TSE was examined using scavenging effect of the stable DPPH free radical [14]. The plant extracts were diluted using twofold dilution to obtain concentrations of 0-10 mg/mL. DPPH of 0.05 mM was prepared with methanol as blank. A volume of 100 *μ*L of DPPH was added to 100 *μ*L of each extract. Ascorbic acid was used as the standard with concentrations ranging from 0 to 10 mg/mL and distilled water as the blank. Negative controls were included. After 20 minutes of incubation the absorbance was measured using a Plate reader (Synergy H1, USA) at a wavelength of 517 nm. Triplicate experiments were performed. The radical scavenging activity of the stem and leaf extracts was expressed as percentage of inhibition using the formula:

(1) Percent%  inhibition  of  DPPH  activity=AB−AAAB×100

where A_A_ and A_B_ represent the absorbance values of the test and of the blank, respectively. A graph of percentage of inhibition versus concentration was used to determine the concentration of the sample required for 50% inhibition (EC_50_).

### 2.5. Determination of Total Phenolic Content (TPC)

The total phenolic content of the fractions was assessed by Folin-Ciocalteu (FC) procedure [15] with some modifications. Gallic acid was used as the standard. Approximately, 50 *μ*L of extract was mixed with 3 mL of distilled water and 250 *μ*L of FC reagent. The mixture was allowed to stand for 5 minutes, and then 750 *μ*L of 20% Na_2_CO_3_ was added. The resulting mixture was vigorously vortexed for 2 min and then incubated for 30 min at room temperature. The absorbance of the solution was measured at 760 nm using a UV-Vis Spectrophotometer (Shimadzu 1201, Japan). All determinations were performed in triplicate. Gallic acid (0.2 mg/mL, 0.4 mg/mL, 0.6 mg/mL, 0.8 mg/mL, and 1 mg/mL) was used as standard to prepare a calibration curve, from which polyphenolic content in terms of the gallic acid equivalent in one gram of each extract was determined. The regression equation y = 0.1x + 0.0032 with a regression factor R^2^ = 0.9976 was obtained. The total phenolic content was calculated from the calibration curve and final results were expressed as mg GAE/100g DM.

### 2.6. Determination of Total Flavonoid Content (TFC)

The aluminium chloride colorimetric assay method [16] was employed to evaluate total flavonoid content (TFC) using quercetin as standard. An aliquot of 500 *μ*L of extract and fractions was mixed with 1.5 mL of 99.9% ethanol (EtOH), 100 *μ*l of 1 M potassium acetate, 100 *μ*L of 10% aluminium chloride, and 3 mL of distilled water. The mixture was shaken vigorously and left to stand in the dark at room temperature. The resulting mixtures were incubated for 30 min at room temperature, and corresponding absorbance was measured at 415 nm. All determinations were carried out in triplicate. A standard calibration curve was constructed using quercetin standard solutions of 20, 40, 60, 80, 100, and 120 *μ*g/mL. 500 *μ*L of each standard was treated in the same manner as the samples above, and a calibration linear regression equation y = 0.0058x - 0.0032 was generated with R2 = 0.9951. Flavonoid content of each extract was determined from the curve and the results were recalculated and expressed as *μ*g QE/100g DM.

### 2.7. Determination of Total Tannins (TT)

The amount of tannins in plant extract and fractions was determined by Folin-Ciocalteu method with slight modifications [15]. 100 uL of the sample extract and fractions was added to 5 mL of distilled water, 500 *μ*L of Folin-Ciocalteu reagent, and 1 mL of 35% Na_2_CO_3_ solution. The mixture was shaken well and kept at room temperature for 30 min. A set of reference standard solutions of gallic acid (0.2, 0.4, 0.6, 0.8, and 1 mg/mL) were prepared in the same manner as described earlier. Absorbance for test and standard solutions was measured against the blank at 725 nm with an UV/Visible Spectrophotometer. The total tannins content was determined from calibration curve: y = 0.1173x + 0.0086, R^2^ = 0.9952, and the results expressed tannin content in terms of mg of GAE /100g DM.

### 2.8. Heavy Metal Analysis

About 1 g of each of the extracts was weighed into a 50 ml digestion tube. The samples were subjected to wet digestion. One mL of H_2_O, 2 mL of HCl, 5 mL of 1:1 HNO_3_:HClO_4_, and 2 mL of H_2_SO_4_ were added and allowed to stand for 20 mins. A temperature of 150°C was regulated to allow the samples to be heated in a digestion block. The digested samples were cooled and further diluted with 50 mL of distilled water. The digests were then analysed for the levels of heavy metals using Atomic Absorption Spectrophotometer (AAS). The metals included lead, copper, cadmium, nickel, zinc, and iron.

### 2.9. UV-VIS and Fourier-Transformed Infrared Spectrophotometry (FT-IR-S) Analyses

About 10 mg/mL of fractions of TSE were diluted in a ratio 1:10 using respective solvents and were further analysed at a wavelength ranging from 200 to 450 nm using a double beam Ultraviolet-Visible Spectrophotometer (Perkin Elmer, USA). The peaks showing the maximum wavelength were recorded. The functional group analyses were performed on the fractions using the FT-IR (Perkin Elmer, USA).

### 2.10. Animals

Albino rats (120-150 g) and mice (20-25 g) were used for the study. They were obtained from the animal facility of the University of Ghana Medical School, Korle Bu, Ghana, and kept at the animal holding facility of the Department of Biochemistry and Biotechnology, KNUST, Kumasi. The animals were kept in aluminium cages bedded with sawdust. The animals had free access to feed (Mash, Agricare, Kumasi, Ghana) and freshly prepared distilled water ad libitum prior to commencement of study to acclimatize to laboratory conditions. They were marked exclusively on their tails using permanent markers for easy identification. All studies were performed according to the guidelines of the Committee for the Purpose of Control and Supervision of Experiment on Animals (CPCSEA, New Delhi, India) and Guide for Care and Use of Laboratory Animals [17].

### 2.11. Acute Oral Toxicity Assessment

Albino mice of either sexes were used for the acute oral toxicity studies. The animals were put into five groups with three animals each: one control group and four treated groups. After an overnight fast, the control group received sterile distilled water while each treated group received 100, 1000, 2500, and 5000 mg/kg b.wt administered orally with the aid of a feeding needle connected to syringe at stated doses dissolved in appropriate volume of sterile distilled water. Doses were selected based on the fixed dose method [18]. The animals were observed for signs of toxicity and mortality for the first critical 4 hours and thereafter daily for 7 days. The oral median lethal dose (LD_50_) was calculated as the geometric mean of dose that caused 0% and 100% mortality, respectively. This was used to guide the selection of three doses (100, 250, and 500 mg/kg b.wt) for subchronic toxicity studies [19].

### 2.12. Subchronic Toxicity Studies

The subchronic toxicity studies were carried out using methods previously described [19]. Briefly, 12 males and 12 female albino rats were divided into 4 groups with 3 rats in each group. A 12-hour fast was allowed before the start of treatment. For each sex, group I served as the vehicle control and received 1 mL/g b.wt freshly prepared distilled water daily while groups II, III, and IV were administered 100, 250, and 500 mg extract /kg b.wt. daily in appropriate volume of distilled water for 28 days. The animals were treated with the extract once daily and observed for signs of toxicity.

### 2.13. Effect of Treatment on Body Weight

Rats were weighed on the first day (D0) and, thereafter, at the end of every four days using a mass balance. The percent change in body weight was calculated using the formula

(2) Percent  Change  in  Body  Weight=Weightn−Weight0Weight0×100%

where Weight_n_ is the weight on D4, D8, D12, D16, D20, D24, and D28 and Weight_o_ is the weight on the first day (D0).

### 2.14. Effect of Treatment on Haematological Parameters of Animals

At the end of the experiment period, animals were fasted overnight and exposed to light ether anaesthetization. Incisions were then made at the cervical regions using sterile blade and blood collected into EDTA tubes for haematology analyses using Sysmex Haematology System (USA). The parameters, haemoglobin concentration (HGB), red blood cell (RBC) count, platelet count, white blood cells, lymphocytes, haematocrit, mean corpuscular volume (MCV), mean corpuscular haemoglobin (MCH), mean corpuscular haemoglobin concentration (MCHC), red cell distribution width (RDW), plateletcrit, platelet distribution width (PDW), and platelet larger cell ratio (P-LCR), were determined.

### 2.15. Effect of Treatment on Some Biochemical Parameters of Animals

Blood was collected into gel activated tubes, left to clot, and centrifuged for 10 minutes at 3500 rpm. The serum obtained was analysed for the levels of Alanine Aminotransferase (ALT), Aspartate Aminotransferase (AST), total bilirubin, total cholesterol (TC), high density lipoproteins (HDL), low density lipoproteins (LDL), Triglycerides (TG), creatinine, urea, and fasting blood glucose using the Selectra E (Vital Scientific, Japan) and reagents from ELITECH (France).

### 2.16. Effect of Treatment on Absolute and Relative Organ Weights

Major body organs which include liver, kidney, heart, stomach, spleen, lung, testes (male), and uterus (female) were excised and washed with buffered saline solution, blotted dry, observed macroscopically, and weighed to obtain the Absolute Organ Weight (AOW). The Relative Organ Weight of each organ was calculated using the following formula.

(3) Relative  Organ  Weight=Absolute  Organ  WeightBody  Weight  at  Sacrifice×100%

### 2.17. Statistical Analysis

Experimental results were expressed as mean ± SEM. Differences in mean were assessed using one-way ANOVA followed by the Tukey's multiple comparison test at significance level of p<0.05. All data was evaluated using the GraphPad Prism 6 for Windows.

## 3. Results

### 3.1. Phytochemical Constituents of* T. stans*

The presence (+) or absence (-) of phytochemical constituents of the raw plant material and the hydroethanolic extract of the leaves is shown in Table 1. The plant and extract were absent with sterols.

### 3.2. DPPH Percentage (%) Scavenging Activity of Fractions

**Figure 1:**
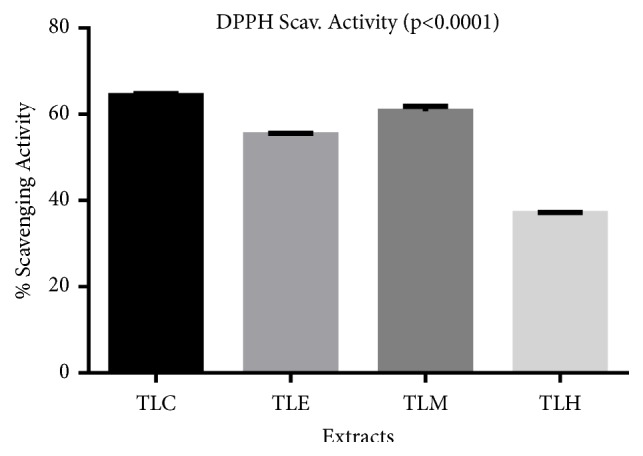
Percentage scavenging activity of crude (TLC), ethyl acetate (TLE), methanol (TLM), and hydro (TLH) fractions of* T. stans*.

Figure 1 shows the DPPH scavenging effect of the crude extract and fractions. The crude extracts (TLC) had the highest activity while the hydro fractions (TLH) had the least percentage.

### 3.3. Total Phenols, Tannins, and Flavonoid of* T. stans* Crude Extract and Fractions

**Figure 2:**
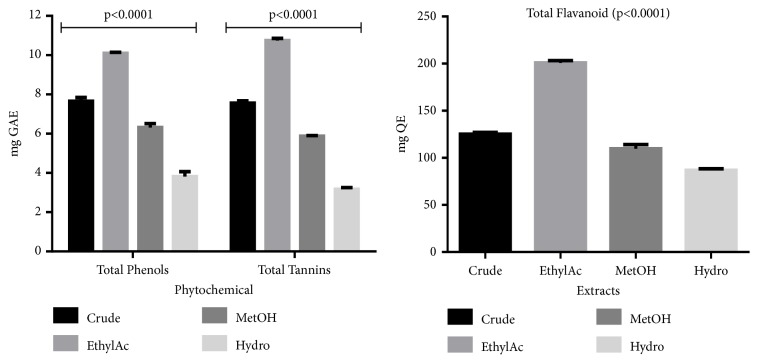
Total phenols, tannins, and flavonoid content of crude and fractions of* T. stans*.

Figure 2 shows the total phenol, tannins, and flavonoid content of crude extract and fractions of* T. stans*. This was based on the standard curves generated for specific standards. The ethyl acetate fraction was the richest in phenols, tannins, and flavonoids while the methanol was the lowest.

### 3.4. Heavy Metal Analysis

**Figure 3:**
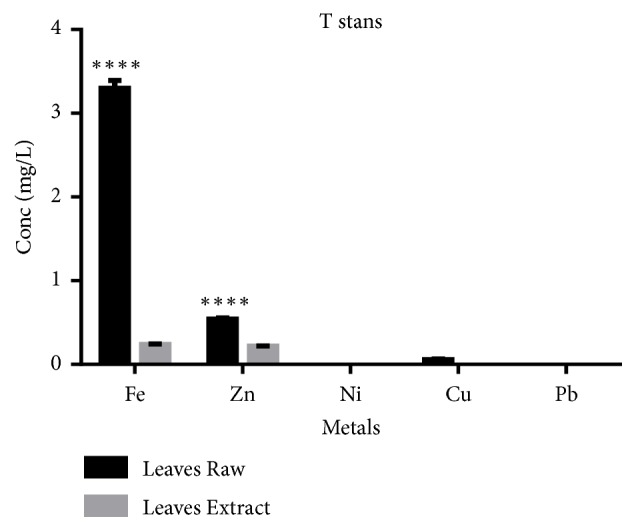
Heavy metal analysis of raw leaves and crude.

Figure 3 shows the metal content of raw plant material and crude extract. The raw leaves gave a high concentration of iron (Fe) as compared to the extract. The zinc in raw leaves also gave a high concentration as compared to the extract. Nickel (Ni) and lead (Pb) were below detection limit (0.0001 ppm).

### 3.5. UV-VIS Spectrophotometry

**Figure 4:**
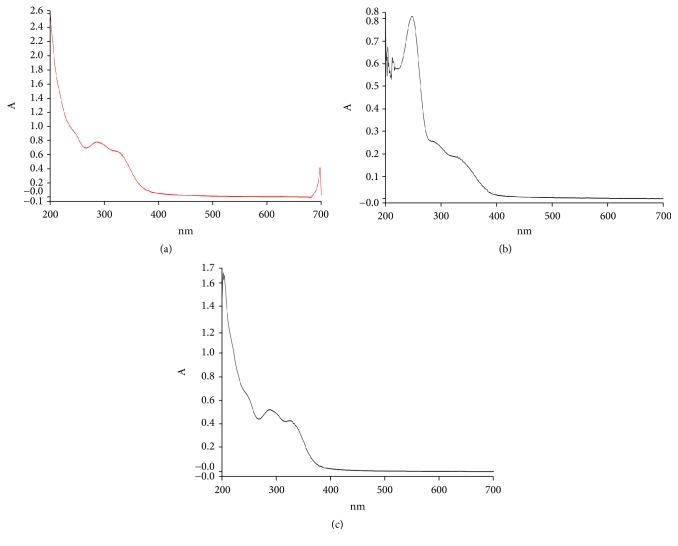
UV-VIS spectrum of hydro (a), ethyl acetate (b), and methanol (c) fraction of* T. stan*s.

The intensity and peak of the fractions of* T. stans *are as shown in Figure 4. The hydro fraction had four (4) intense peaks (697.4 nm–0.4225; 322.6 nm–0,6033; 327.9 nm–0,6406; 285.4 nm–0.7860); the petroleum ether fraction had 5 peaks (247.4 nm–0.8114; 204.0 nm–0.6758; 201.9 nm–0.6542; 212.2 nm–0.6264; 205.9 nm–0.6062), and the methanol extract had 3 peaks (287.9 nm–0.5222; 204.0 nm–1.658; 202.0 nm–1.680).

### 3.6. FT-IR Analyses on the Fractions of* T. stans*

**Figure 5:**
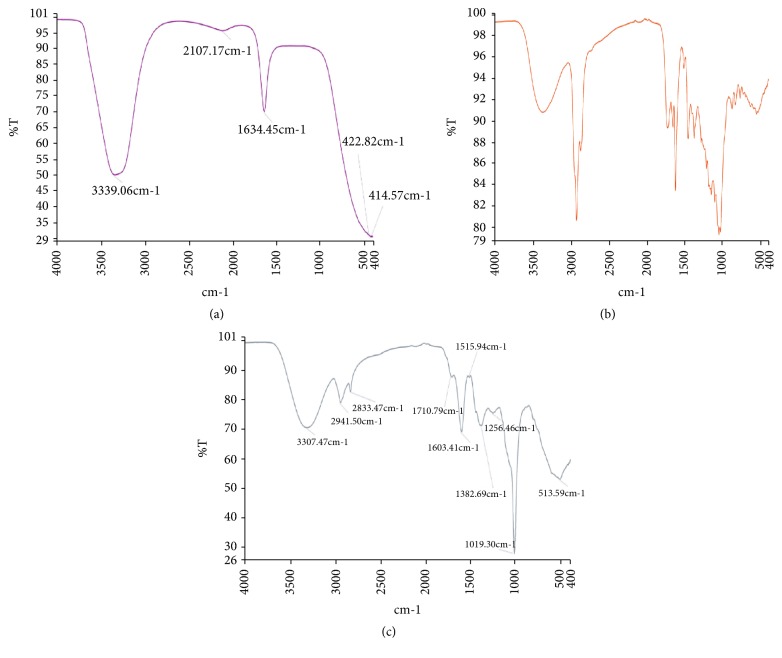
FT-IR spectra on hydro (a), ethyl acetate (b), and methanol fractions of* T. stans*.

FT-IR spectra (% transmittance over wavenumber) of* T. stans* fractions are as shown in Figure 5 and Table 2. All fractions were rich in phenols, carbonyl compounds, and other compounds.

### 3.7. Acute Toxicity of TSE

Hydroethanolic leaf extract of* T. stans *had no mortality or significant behavioural changes up to 5000 mg/kg b.wt. in mice. Therefore, the LD_50_ is estimated at LD_50_≥ 5000 mg/kg b.wt. in mice. It is therefore considered as safe.

### 3.8. Subchronic Toxicity of TSE

*Tecoma stans *aqueous-ethanolic extract (TSE) was administered at doses of 100 mg/kg, 250 mg/kg, and 500 mg/kg b.wt. In general, no adverse clinical or behavioural observations were made after the first 4 critical hours. No death or obvious clinical signs were observed in any group throughout the study period.

### 3.9. Effect of Treatment on Percentage Change in Body Weight of Animals

**Figure 6:**
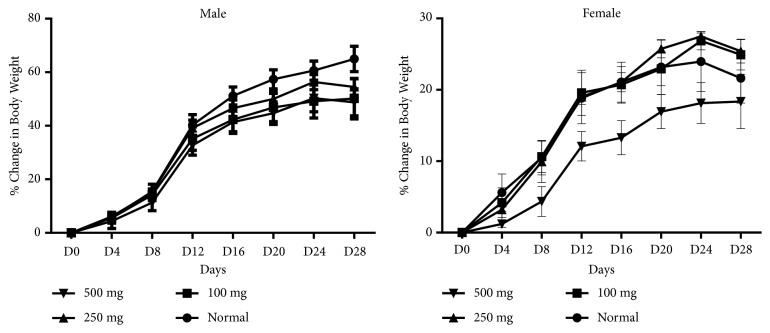
Effect of treatment on body weight of male and female animals.

Figure 6 shows the percentage of body weight of male and female animals. Significant increases in weight was observed at all doses from Day 0 to Day 28 in both male and female rats. Throughout the study, there was no significant change in male rats as compared with the normal. Female rats administered 250 mg/kg b.wt. showed the highest increase in body weight compared to the normal followed by the 100 mg/kg b.wt while there were no significant change in the 500 mg/kg b.wt.

### 3.10. Effect of Treatment on Relative Organ Weight

Table 3 shows the effect of treatment on Relative Organ Weight of animals. No significant changes were observed in all organs at all doses among male and female rats.

### 3.11. Effect of Treatment on Haematological Indices of Animals

From Table 4, there was a significant increase in WBC count in male animals treated with 500 mg (p<0.01) compared with the other groups and a significant decrease in WBC count in female rats treated with 100 mg (p<0.05) and 250 mg (p<0.01). Also, significant decrease in %PCT in male rats was observed at 250 mg (p<0.05) and significant decrease in P-LCR in female rats at 100 mg (p<0.05) dose.

### 3.12. Effect of Treatment on Biochemical Parameters of Animals

As shown in Table 5, significant increase in AST levels was observed at 500 mg (p<0.01) in the male rats and AST/ALT ratio showed a significant increase in female rats at 500 mg (p<0.05) compared to the normal. Significant increase in TBil was observed at 500 mg (p<0.001) and IBil at 500 mg (p<0.01). Creatinine increased significantly at 250 mg (p<0.01) compared to the normal in both male and female rats. FBG showed significant increase in male rats at 500 mg (p<0.01), and a significant increase was observed in female rats administered 500 mg (p<0.001) compared to the normal.

## 4. Discussion

Awareness of the chemical components of plants is important for the discovery of therapeutic bioactive compounds found in medicinal plant as well as the synthesis of new drugs [20]. The presence of glycosides, tannins, flavonoids, alkaloids, saponins, and coumarins in the raw leaves and extract can have synergistic effect influencing the efficacy of the extract to produce a desired or intended pharmacological effect reported [21]. This was further confirmed by the UV-Vis and FT-IR spectra, showing varying functional groups in the fractions

DPPH percentage scavenging activity is the percentage for a compound to mop out free radicals released in a body. This is normally calculated to find out the potency of a plant material in relation to its antioxidant activity. Based on the observations, the crude hydroethanolic extract had the highest scavenging activity (64.32%), thus contributing to the ameliorative effect described for some disease conditions [22]. This scavenging activity could be caused by the phenols and flavonoid measured in the extracts

The accumulation of heavy metals has been reported in some medicinal plants [23–25], which could explain some associated toxicity. To this effect, some heavy metals were assessed in the extract. Nickel and lead were not detected; however, the extract was rich in iron and zinc that could enhance some biochemical effects.

Acute toxicity study provides initial information on the mode of toxic action of an agent, acts as the basis for classification and labelling, and helps in deciding the dose of novel compounds in animal studies. In the current study, the LD_50_≥ 5000 mg/kg b.wt was observed, indicating the extract to be safe at the acute level. Subchronic toxicity provides information on dosage level safe for administration and targeted organ toxicity and identifies observable adverse effect that may affect the average lifespan of experimental animals. Consequently, in this study, TSE was evaluated in rats at doses 100, 250, and 500 mg/kg b.wt. for 28 days. The body weight changes, a sensitive indicator of general health status of animals [26], was observed. TSE did not interfere with the normal metabolism of animals but rather improved appetite and food utilisation.

Organ weight determination in subchronic toxicity serves as a sensitive indicator of chemical changes to organs [27]. It also indicates which organs are being targeted in case of any major toxicities as well as accumulation of the test substance [28]. In this study, no serration or damages were observed on harvested organs indicating any subchronic toxicity on the organs observed.

The haematological parameters can be used to determine the blood relating functions of plant extract. The TSE indicated a nonsignificant difference on the RBC count and its indices except a significant rise in the %HCT in the female at 250 mg. This may reflect an absolute increase in the number of erythrocytes or a decrease in plasma volume in conditions such as dehydration [29]. In general, it can be said that the results obtained do not affect erythropoiesis, morphology, or osmotic fragility of red blood cells [30]. In the WBCs, the male rats showed significant increase in WBC level at 500 mg indicating an increase in immunity when compared to the other groups and the normal. Also, female rats showed significant decrease in WBCs at 100 mg and 250 mg doses when compared to the 500 mg and the normal. This on the other hand indicates decline in immunity. This suggests that the extract at the small doses of 100 mg and 250 mg could cause immunological defects in the female rats which render them vulnerable to infections since WBCs are the first line of cellular defence that respond to infectious agents, tissue injury, or any inflammation. Significant decrease in platelet count was observed in male rats treated with 250 mg and a significant decrease in plateletcrit (%PCT) in male rats treated with 250 mg, with possible effect in preventing platelet aggregation, a prominent effect in disease condition [31].

Abnormal levels of hepatic enzymes biomarkers usually refer to high levels of the enzymes in blood, which is practically an indicator of a damaged liver. Conversely, low biomarker levels usually have no clinical significance and are associated with a healthy liver [32]. In this study, significant increase in AST levels was observed at 500 mg in the male rats. This suggests that, at higher doses, the TSE compromises the liver function which may lead to damage of the liver as it increased gradually from the 100 mg to 500 mg when compared to the normal. The impaired liver function at 500 mg/kg was further corroborated by the increased total bilirubin (TBil) levels. With respect to renal function, high clearance of creatinine and urea from the blood is significant for proper functioning kidneys. In this study, significant increase in creatinine levels was observed at 250 mg compared with the normal in both male and female rats. The high level shows renal disease and muscle wasting disorders [33].

The study also revealed significant increase in FBG observed in male and female rats administered 500 mg compared to the normal. High FBG is usually related to hormone action. These hormones include insulin, which facilitates the movement of glucose from food ingested into the body cells for energy production, and glucagon, which breaks down glycogen stored in the liver and muscles, releasing glucose to produce energy when levels from food are low or not available [34]. It is very common to have a high glucose level after a fast due to the action of glucagon. Therefore, the significantly high fasting blood glucose at 500 mg/kg could indicate some form of hormonal imbalance at the highest dose affecting the suitable breakdown of glucose. The determination of lactate dehydrogenase (LDH) activity has a wide variety of clinical uses. As an intracellular enzyme, its increase indicates tissue damage with its consequent release to the blood stream. In this study, no significant change was observed in the LDH levels at all doses in both male and female rats. This result indicates that there was no significant damage to tissues and major organs in both sexes.

## 5. Conclusion

This study on TSE confirms the presence of the phytochemicals responsible for its pharmacological activities and that it can serve as a therapeutic agent. The study also showed that TSE had no impairment on the nutritional benefits of the experimental rats, and it did not pose any deleterious effects to major organs of the body. However, significant decrease in platelets levels, decrease in WBCs, and increase in blood glucose were observed at higher doses. These findings show that* Tecoma stans *leaves have no prospective adverse or toxicological effect and suggests that it is safe with controlled use.

## Figures and Tables

**Table 1:** Phytoconstituents of raw leaf and hydroethanolic extract of *T. stans*.

Phytoconstituents	Raw Leaf	TSE
Glycosides	+	+
Tannins	+	+
Flavonoids	+	+
Alkaloids	+	-
Saponins	+	+
Coumarins	+	+
Sterols	-	-

**Table 2:** Functional groups present in fractions of *T. stans*.

Wave numbers (cm^−1^)	Y (%T)	Bond	Assignments
*Hydro fraction*			
3339.06	50.56	O-H stretch, H bonded	Alcohols, phenols
2107.17	95.75	C≡C stretch	Alkynes
1634.45	70.36	C=O stretch	Carbonyls (general), *α*, *β*-unsaturated aldehydes, ketones
422.82	31.11	Unknown	Unknown
414.57	31.14	Unknown	Unknown
*Ethyl acetate fraction*			
3373.96	90.79	O-H stretch, H-bonded	Alcohols, phenols
2926.73	80.64	O-H stretch	Carboxylic acids
2872.58	87.17	N-H stretch	1°, 2° amines, amides
1726.16	89.32	C=O stretch	*α*,*β*-aldehydes, ketones, saturated aliphatic acids
1655.64	89.42	C=C stretch	Alkenes
1457.11	88.34	C-H bend	Alkanes
1377.97	88.37	Unknown	Unknown
1215.54	85.51	C-H wag	Alkyl halides
1153.59	83.07	C-O stretch	Alcohols, carboxylic acids, esters, ethers
837.67	91.50	C-Cl stretch	Alkyl halides
773.57	92.13	C-Cl stretch	Alkyl halides
556.54	90.63	C-Br stretch	Alkyl halides
*Methanol fraction*			
3307.47	70.71	O-H stretch, free hydroxyl	Alcohols, phenols
2941.5	78.95	O-H stretch	Carboxylic acids
2833.47	82.61	C-H stretch	Alkanes
1710.79	87.57	C=O stretch	*α*.*β*-unsaturated aldehydes, ketones.
1603.41	69.06	C-C stretch (in ring)	Aromatics
1515.94	87.45	N-O asymmetric stretch	Nitro compounds
1382.69	71.15	C-H rock	Alkanes
1256.46	75.39	C-N stretch	Aliphatic amines
1019.30	27.61	C-O stretch	Alcohols, carboxylic acids, esters, ethers.
513.59	52.92	C-Br stretch	Alkyl halides

**Table 3:** Effect of TSE on the Relative Organ Weight (Row) of Animals.

	Normal	100 mg	250 mg	500 mg
*Male*				
Liver	3.18±0.22	3.02±0.07	3.08±0.16	3.25±0.05
Kidneys	0.55±0.02	0.58±0.03	0.56±0.04	0.55±0.01
Testes	1.16±0.01	1.27±0.08	1.17±0.01	1.04±0.10
Lung	0.63±0.07	0.82±0.02	0.77±0.07	0.68±0.02
Stomach	0.60±0.03	0.69±0.09	0.58±0.02	0.61±0.01
Heart	0.32±0.02	0.34±0.02	0.32±0.03	0.30±0.01
Spleen	0.19±0.01	0.18±0.00	0.18±0.01	0.19±0.01

*Female*				
Liver	2.95±0.10	3.16±0.11	2.78±0.09	2.83±0.11
Kidneys	0.58±0.00	0.58±0.00	0.58±0.01	0.61±0.02
Lung	0.73±0.07	0.84±0.01	0.81±0.15	0.55±0.04
Stomach	0.65±0.03	0.63±0.04	0.63±0.01	0.55±0.02
Heart	0.35±0.02	0.31±0.01	0.34±0.01	0.33±0.10
Uterus	0.26±0.03	0.27±0.04	0.24±0.02	0.19±0.02
Spleen	0.23±0.01	0.23±0.01	0.22±0.01	0.20±0.01

*Mean±SEM (n=3)*.

**Table 4:** Effect of TSE on haematological parameter of animals.

	Normal	100 mg	250 mg	500 mg
*Male*				

WBCx10^3^/*μ*L	6.10±0.50	5.83±0.92	6.03±0.35	8.57±0.87*∗∗∗∗*
RBCx10^6^/*μ*L	8.09±0.46	7.78±0.19	8.24±0.17	8.06±0.17
HGB g/dL	14.10±0.66	14.07±0.12	14.83±0.17	14.47±0.20
HCT %	58.26±3.44	56.53±1.56	60.50±0.75	57.67±1.03
MCV/ Fl	72.03±0.74	72.60±0.21	73.47±1.36	71.57±0.32
LYM%	61.20±3.91	62.77±0.84	66.93±0.92	65.26±1.46
MCHC g/Dl	24.23±0.35	24.93±0.72	24.50±0.40	21.70±3.36
RDW-SD/Fl	43.80±1.27	47.66±3.25	46.87±0.95	44.33±1.17
MCH pg	17.43±0.20	18.10±0.49	18.03±0.20	15.53±2.47
RDW-CV/%	15.16±0.29	16.76±1.64	16.03±0.38	15.43±0.42
PDW/Fl	10.23±0.23	9.87±0.40	10.23±0.75	10.07±0.14
MPV/Fl	8.40±0.11	8.20±0.17	8.30±0.42	8.23±0.09
P-LCR/%	13.56±1.01	12.13±1.02	13.67±2.67	13.03±0.28
PLT 10∧3/*μ*l	1058.67±80.76	969.33±163.24	750.00±62.55	1014.00±189.94
PCT/%	0.89±0.06	0.79±0.13	0.62±0.03*∗*	0.84±0.16

*Female*				

WBC × 10∧3/*μ*l	7.30±0.30	4.97±0.33	5.03±0.35	7.40±0.26*∗∗∗∗*
RBC × 10∧6/*μ*L	7.20±0.06	7.35±0.06	7.73±0.06	7.16±0.13
HGB g/Dl	13.43±0.09	13.60±0.26	14.03±0.26	13.30±0.36
HCT %	52.07±0.88	53.17±0.82	56.53±1.20*∗*	50.97±1.57
MCV/ Fl	72.30±0.60	72.33±0.49	73.16±1.69	71.13±0.96
LYM%	63.47±0.48	66.47±1.53	64.40±0.44	66.83±0.87
RDW-SD/Fl	39.83±0.75	41.07±1.88	41.17±1.22	39.33±0.39
MCH pg	18.63±0.12	18.47±0.23	18.17±0.34	18.57±0.17
P-LCR/%	11.90±0.56	10.07±0.39	12.70±1.00	13.27±0.75
RDW-CV/%	13.40±0.25	13.90±0.87	13.67±0.17	13.43±0.37
PDW/Fl	9.83±0.19	9.33±0.17	10.03±0.19	10.17±0.23
MPV/Fl	8.13±0.09	7.83±0.03	8.20±0.15	8.23±0.09
PLT 10∧3/*μ*l	1323.00±156.52	1038.67±73.49	1105.33±105.49	1083.67±139.71
PCT(%)	0.81±1.20	0.73±0.79	1.05±0.93	0.76±1.15

Mean±SEM (n=3); statistical significance; *∗*p<0.5, *∗∗∗∗*p<0.001 compared among the treated and normal groups.

**Table 5:** Effect of TSE on the biochemical parameters of animals.

	Normal	100 mg	250 mg	500 mg
Male				

ALT (U/L)	63.87±5.27	48.70±3.45	65.57±0.98	71.50±2.26
AST (U/L)	148.00±2.55	153.43±2.48	169.30±6.50	189.47±15.60*∗∗*
AST/ALT	0.67±0.52	1.47±0.28	0.83±0.44	1.67±0.29
TBil (*µ*mol/L)	1.63±0.55	2.96±0.88	1.85±0.42	2.68±0.27
DBil (*µ*mol/L)	0.95±0.05	1.49±0.65	0.98±0.03	1.04±0.17
IBil (*µ*mol/L)	0.67±0.52	1.47±0.28	0.83±0.44	1.67±0.29
Creat (*µ*mol/L)	28.50±6.22	24.27±1.19	49.67±9.19*∗∗*	23.03±4.95
Urea (mmol/l)	11.04±0.85	8.09±1.25	8.95±0.58	9.26±1.02
K (mmol/L)	7.30±0.45	6.27±0.19	7.00±0.35	6.83±0.44
Na (mmol/L)	143.00±0.43	142.43±0.55	142.90±0.42	143.80±1.95
Cl (mmol/L)	105.57±0.53	102.77±0.67	104.80±0.87	106.43±1.05
TChol (mmol/L)	1.87±0.07	2.13±0.26	2.11±0.21	2.04±0.09
Trigs (mmol/L)	1.19±0.15	1.35±0.29	1.14±0.12	1.33±0.16
VLDL (mmol/L)	0.53±0.09	0.60±0.15	0.50±0.06	0.60±0.10
HDL (mmol/L)	0.58±0.02	0.79±0.07	0.53±0.07	0.59±0.09
LDL (mmol/L)	0.74±0.03	0.73±0.31	1.06±0.16	0.85±0.11
Glucose (mmol/L)	1.72±0.32	2.48±0.21	3.02±0.33	4.60±0.77
LDH (U/L)	4617.76±101.23	4521.53±404.56	4340.33±184.88	4462.07±649.88

Female				

ALT (U/L)	56.70±4.22	49.97±2.98	56.63±3.86	46.20±2.46
AST (U/L)	149.77±8.24	143.07±5.01	144.37±19.11	169.17±10.95
AST/ALT	2.67±0.18	2.87±0.09	2.57±0.26	3.67±0.14
TBil (*µ*mol/L)	1.36±0.27	1.91±0.43	1.68±0.21	3.31±0.46*∗∗∗*
DBil (*µ*mol/L)	0.93±0.11	1.45±0.24	1.06±0.09	1.22±0.21
IBil (*µ*mol/L)	0.43±0.18	0.80±0.40	0.63±0.02	2.07±0.42
Creat (*µ*mol/L)	28.50±0.66	25.80±5.05	41.70±5.03*∗∗*	22.87±3.33
Urea (mmol/l)	10.61±0.46	12.61±0.82	11.64±1.20	7.55±1.45
K (mmol/L)	7.07±0.29	7.13±1.24	7.20±0.40	5.80±0.21
Na (mmol/L)	142.73±0.43	142.77±0.58	141.20±0.93	145.47±1.44
Cl (mmol/L)	106.33±0.80	106.97±1.30	105.97±0.81	109.13±1.33
TChol (mmol/L)	2.15±0.03	2.45±0.07	2.36±0.06	2.11±0.17
Trigs (mmol/L)	1.27±0.22	1.07±0.30	0.96±0.29	1.62±0.26
VLDL (mmol/L)	0.57±0.09	0.50±0.15	0.43±0.12	0.73±0.12
HDL (mmol/L)	0.87±0.11	0.71±0.27	0.76±0.08	0.43±0.04
LDL (mmol/L)	0.70±0.12	1.25±0.20	1.16±0.15	0.95±0.17
Glucose (mmol/L)	1.20±0.10	1.42±0.48	1.26±0.15	5.09±0.34
LDH	4052.10±101.65	3860.33±90.55	4039.20±239.98	3301.40±638.74

Mean±SEM (n=3); statistical significance; *∗*p<0.5, *∗∗*p<0.01, and *∗∗∗*p<0.001 compared among the treated and normal groups.

## Data Availability

Supporting data for the findings of this study are available upon request from the corresponding author.
